# Thirty-eighth Commencement of the Medical Department of the University of Buffalo

**Published:** 1884-03

**Authors:** 


### THIRTY-EIGHTH COMMENCEMENT OF THE MEDICAL DEPARTMENT OF THE UNIVERSITY OF BUFFALO.

The annual commencement exercises of the Medical Universily of Buffalo were held in this city Feb. 26, 1884. The meetings of the Alumni Association were also held during the day in the college buildings. The following members of the Alumni Association were present during the day: Dr. E. N. Brush, of Utica, President; Dr. A. M. Barker, of Buffalo, Secretary.

Of the class of—

'47—William Ring, Buffalo.

'48—C. C. Wyckoff, Buffalo; H. Hoyt, Aurora, N. Y.; A. G. Ellenwood, Attica, N. Y.

'51—T. D. Strong, Westfield.

'52—George Abbott, Hamburg.

'53—C. L. Dayton, Buffalo.

'59—W. W. Potter, Buffalo.

'61—F. D. Ritter. Gaines, Pa.

'65—H. Lapp, Clarence.

'66—Conrad Diehl, Buffalo; W. C. Phelps, Buffalo.

'67—E. C. W. O'Brien, Buffalo.

'68—John Dambach, Buffalo.

'69—John H. Taylor, Holley, N. Y.; G. W. Pattison, Buffalo.

'70—C. B. Kibler, Corry, Pa.

'71—D. W. Harrington. John J. Walsh, A. H. Briggs, Buffalo; O. C. Strong, Colden, N. Y.

'72—P. W. Van Peyma, Charles B. Knowlton, F. E. L. Brecht, Buffalo; F. H. Moyer, Moscow, N. Y.

'73—F. E. Dewey, Peterboro; Ambrose Kassan, Bath; Joseph Fowler, D. L. Bailey, Buffalo.

'74—E. N. Brush, Utica; Bernard Bartow, Buffalo.

'75—W. B. Cottier, Buffalo.

'76—C. H. Wetzel, Mrs. Mary B. Moody, A. H. Hubbell, W. J. Packwood, Buffalo.

'77—C. C. Harvey, Dundee, N. Y.; A. M. Barker, Buffalo; W. F. Sheehan, Rochester; I. M. Dike, Jordan, N. Y.; Lee H. Smith, Frank C. Sherwood.

'78—Charles A. Ring, Buffalo Plains.

'79—Frederick Peterson, W. H. Pitt, C. A. Wall, Buffalo.

'80—F. O. Vaughan, Edward Clark, W. C. Barrett, S. H. Warren, Mary Burkea, B. H. Grove, E. C. Waldruff, Buffalo.

'81—John J. Birmingham, Mary E. Runner, Irving M. Snow, Buffalo; W. A. Hobday, Alton, Pa.

'82—J. Frank, Eli H. Long, F. H. Potter, Charles Weil, Willis G. Gregory, J. W. Putnam, Buffalo; D W. Wollaber, Cambria, N. Y.; W. B. McDougall, Spencerport, N. Y.

'83—E. G. Long, Buffalo; B. D. Beidelman, Tunkhannock, Pa.; Mrs. Isabella Stanley, Dnnkirk, N. Y,; F. B. Burroughs, Columbus, Pa.

Honorary—J. B. Coakley.

Dr. W. C. Barrett, Chairman of the Executive Committee, and Dr. C. C. Wyckoff, Treasurer, made their annual report. A committee consisting of Drs. Dewey, Ring, Potter, Walsh, and Diehl, was appointed to nominate officers for the ensuing year. The following is the report of the committee whose nominations were confirmed: After due deliberation we present the following list of officers for the coming year:

President—E. C. W. O'Brien, Buffalo.

First Vice-President—C. Diehl, Buffalo.

Second Vice-President—D. W. Harrington, Buffalo.

Third Vice-President—Mary B. Moody, Buffalo.

Fourth Vice-President—W. F. Sheehan, Rochester.

Fifth Vice-President—Theodore H. Boysen, Buffalo.

Secretary—A. M. Barker, Buffalo.

Trustees—F. W. Abbott, C. C. Wyckoff, William C. Phelps, P. W. Van Peyma, Buffalo: Henry Lapp, Clarence.

Executive Committee—Mhe President of the Association, ex-officio; the Secretery of the Faculty, ex-officio; C. A. Ring, J. W. Putnam, F. H. Potter, Buffalo.

Honorary Members—Roswell Park, Herman Mynter, J. F. King, E. T. Dorland, Buffalo.

After the address of the President, Dr. E. N. Brush, on the general subject of the preparatory education of medical students, an interesting paper on "Nerve Force" which the JOURNAL expects to be able to present to its readers in its next number, was read by Prof. W. H. Pitt, of Buffalo. Prof. Roswell Park next read a practical paper on the radical cure of Hernia. He advocated the method of Czerny of Heidelberg, in which the writer has had considerable experience and which method seems not to have received in this country the favorable notice to which apparently it is entitled. The concluding essay of the session was by Dr. William F. Sheehan, of Rochester, on "Common Defects of House Drainage." This was illustrated by means of the Steriopticon. The subject received very able and interesting treatment and was discussed by Drs. Andrews and Nott. A vote of thanks was given to the several essayists and the meeting adjourned.

The commencement exercises at Concert Hall were held in the evening, the hall being completely filled. Upon the platform were seated the members of the faculty, the curators and members of the council. After music by the orchestra, the commencement exercises were inaugurated by prayer by the Rev. Dr. Van Bokkelen, chaplain of the college.

Prof. Charles Cary presented the class in the following words: "Mr. Chancellor, I take pleasure in presenting this graduating class. They have passed good examinations, and I present them to you that they may receive their degree."

Dr. Rochester, as vice-chancellor, responded and requested the class to arise, lift the right hand, in order that they might take the Hippocratic oath. He swore them to do their duty to their patients, never to reveal the secrets of those who placed themselves in their care, and never to administer medicine for any improper or unlawful purpose.

The degrees were conferred in the usual manner by the vice-chancellor, Prof. Thomas F. Rochester, upon the following:

Ahlstrom, Christian P., Jamestown, N. Y.
Babcook, Marcus F., Hammondsport, N. Y.
Bartholomew, Lorenzo S., Dundee. N. Y.
Bingham, Henry H. Lancaster, N. Y.
Burchard, William W., Dalton, N. Y.
Borland, Wilbur P., Richfield Springs, N. Y.
Brewer, George E., " A. B., Buffalo, N. Y.
Brooks, Mark N., Colden, N. Y.
Campbell, M. Ushe1, Lebanon; N. Y.
Callahan, William C., Buffalo, N. Y.
Caswell, James C., Cassadaga, N. Y.
Connclly, John P., Binghamton, N. Y.
Cross, Irwin M., Olean, N. Y.
Cott, George F., Buffalo, N. Y.
Crowe. Thomas M., Corry, Pa.
Culver, Miss Anna B., Des Moins, Iowa.
Dauser, Earl G., Clarence, N. Y.
Doherty, Edward P., Menram Cook, N. B.
Eaton, Edelburt M., Ulysses, Pa.
French, Wallace J., Wellorsfield, N. Y.
Gardener, William F,, Conewango, N. Y.
Goldberg, Sedmond, Buffalo.
Hammill, Vincent G., Phenix. N. Y.
Harding, Arthur B., Hume, N. Y.
Herrmann, Samuel S., Bradford, Pa.
Johnson, Matthias, Redwing. Minn.
Kelly John George, Bergen, N. Y.
Larzalere, John V., Waterloo, N. Y.
Lyman, Almon H., Hume, N. Y.
Loomis, Boardman J., Buffalo, N. Y.
Matzinger, Herman G. A., Buffalo, N. Y.
McCollum, George D., Ransomville, N. Y.
McDonald, William, Buffalo, N, Y.
McMichael, Jacob D., Buffalo, N. Y.
Millar, Wm. John L., Russell, N. Y.
Mullen, Isaac T., Alexander, N. Y.
Neumann, Theodore, Buffalo, N. Y.
Oliver, William A., Penn Yan, N. Y.
Park, William, Buffalo, N, Y.
Pawling, Thomas H., Bath, N. Y.
Payne, Charles S., Berkshire, N. Y.
Peterson, Elliott M., Jamestown, N. Y.

Pitkin, John T., Buffalo, N. Y.
Porter, James, Urbana, Iowa.
Putnam, William A., Cassadagb, N. Y.
Richardson, Edwin C., Alexander, N. Y.
Ring, William G., Buffalo, N. Y.
Robertson, Charles B., Towlesville, N. Y.
Rochester, Delancey, Buffalo, N. Y.
Rowley, Ransom F., Cuba, N. Y.
Schmitz, M., Wisconsin.
Scofield, Era M., Bemus Point, N. Y.
Seymour, Burton W., Stockton, N. Y.
Shaffner, Edward M., East Ashford, N. Y.
Smith, John W., Buffalo, N. Y.
Snover, Halsey D., Machias, N. Y.
Storr, Elmer G., Edwardsburg, Mich.
Stone, Harry H., Franklinville, Ont.
Tanner, William T., Buffalo, N. Y.
Vanderburg, Frank P., Mt. Vernon, N. Y.
Wisner, Andrew P., Mt. Morris, N. Y.
Woolson, William C., Niagara Falls, N. Y.

Dr. Rochester then announced that two of the graduates had been selected from the excellence of their theses for honorable mention. For the thesis on "Phosphoric Acid in Urine," John T. Pitkin of Buffalo took the first place, and for a thesis on "Morbus Coxarine," Will F. Gardner of Conewango was entitled to the second place. Dr. Rochester further said that the faculty had decided to introduce a new feature in the line of honors for merit, and it was one that was wholly unexpected by the class. The men who stood at the head had been selected as entitled to honorable mention. In this class he read the following:

First place—William McDonald.
Second place—De Lancey Rochester and Frank P. Vandenburg.
Third place—George E. Brewer and M. U. Campbell.
Fourth place—Charles S. Payne and John T. Pitkin.
Fifth place—Edward P. Doherty, Elliott M. Peterson and William A. Pntnam.

The exercises consisted of an address to the graduating class of Prof. Roswell Park, which was well received. The address to the alumni was by the Hon. James O. Putnam, and was one which cannot be too highly praised. We are glad to find that the paper is published in full in the columns of the *Commercial Advertiser*, and we earnestly urge our readers to obtain a copy that they may read this thoughtful and most eloquent address. The exercises concluded with the benediction by the Rev. Van Bokkelen.

The banquet was given at the Genesee, and was without exception the best in all respects which it has been our privilege to attend. The replies to the several toasts were uniformly excellent and we have no hesitation in saying that all departed in the most pleasant frame of mind.

